# A virtual reality-based self-guided training on identification of negative automatic thoughts in healthy adults: a mixed-methods feasibility study

**DOI:** 10.3389/fpsyt.2024.1479207

**Published:** 2024-11-27

**Authors:** Bixi Yang, Chenxi Liao, Yuqing Yang, Binbin Shi, Caidi Zhang, Chunbo Li

**Affiliations:** ^1^ Shanghai Mental Health Center, Shanghai Jiao Tong University School of Medicine, Shanghai, China; ^2^ Shanghai Yangpu District Mental Health Center, Shanghai University of Medicine and Health Sciences, Shanghai, China; ^3^ Shanghai Key Laboratory of Psychotic Disorders, Shanghai Mental Health Center, Shanghai Jiao Tong University School of Medicine, Shanghai, China; ^4^ Institute of Psychology and Behavioral Science, Shanghai Jiao Tong University, Shanghai, China

**Keywords:** virtual reality, cognitive behavioral therapy, cognitive restructuring, negative automatic thoughts, feasibility

## Abstract

**Background:**

Cognitive restructuring (CR) is an evidence-based psychological technique for depression. However, face-to-face CR is not easily accessible. Digital CR interventions often overlook the difficulties individuals experiencing depression encounter in identifying their negative automatic thoughts (NAT), leading to suboptimal outcomes. Virtual Reality (VR) has potential advantages in assisting the identification of NAT in CR intervention.

**Objective:**

The aim of this preliminary feasibility study is to assess the efficacy, acceptability and safety of a VR-based self-guided training on the identification of negative automatic thoughts (VR-STINAT) for depression, as well as to evaluate the user experience.

**Methods:**

In a mixed methods study, 20 healthy participants underwent VR-STINAT and completed a semi-structured interview, followed by post-training homework. The VR-STINAT includes three modules: psychological education, NAT identification training in VR scenarios, and practice in personally experienced scenarios. Effectiveness was measured via Thought Record Skills Assessment (TRSA) of homework and Cognitive Therapy Awareness Scale (CTAS). Acceptability was measured using an adapted Technology Acceptance Model and duration of training. Safety was measured via Simulator Sickness Questionnaire and self-reported negative emotions. Qualitative material was analyzed using thematic analysis.

**Results:**

The VR-STINAT was acceptable, with an average rating of 80.68%. The accuracy of NAT identification in TRSA reached 84.55%, and CTAS correctness reached 76.67%. The majority of participants experienced minimal or no side effects, although a few (10%, 2/20) reported relatively severe fatigue and craniofacial pain. Thematic analysis reviewed four themes: effectiveness, acceptability, advantages of VR, difficulties in use and suggestions for improvement. Most participants indicated that they’ve learned how to identify their NAT through VR-STINAT (85%, 17/20), which was engaging (90%, 18/20) and easy to use (60%, 12/20).

**Conclusion:**

This study provides preliminary evidence that self-guided training for the identification of negative automatic thoughts related to depression using VR is feasible. Future studies are needed to compare the efficacy of VR with other intervention modalities in people with depression.

## 1 Introduction

Cognitive restructuring (CR) is an evidence-based psychological technique for depression (1). Automatic thoughts (AT), the most accessible levels of cognition and generally the first to be targeted in CR, refer to individuals’ immediate interpretations of specific situations, which occur spontaneously in consciousness. AT can occur so quickly that individuals may not always be fully aware of them, yet they can significantly impact their emotions (2). Identification of negative automatic thoughts (NAT) that trigger intense emotions is the initial step of CR, followed by evaluation for veracity and functionality before modification.

However, individuals experiencing depression may encounter challenges in identifying their own NAT due to their cognitive and psychological characteristics. First, individuals experiencing depression often experience alexithymia (3), making it difficult for them to adequately identify and describe their emotions and thoughts. Secondly, depression has been associated with over general autobiographical memory, manifested by a reduction in the quantity of specific memories (4). This impedes the ability of individuals experiencing depression to identify previous NAT through recollection. Moreover, experiential avoidance, including behavioral and cognitive avoidance, serves as a coping mechanism among individuals experiencing depression to avoid negative emotions, but this can negatively impact NAT identification (5, 6).

In face-to-face CR therapy, to help individuals experiencing depression to identify their NAT, therapists often employ techniques such as role playing and guided imagery (6). However, individuals experiencing depression may withhold their genuine NAT due to concerns about the therapist’s response. In addition, the current accessibility of professional psychotherapy is limited (7). In China, only 9.5% of individuals experiencing depression have received mental health services, and the proportion of those considered to have received adequate treatment (antidepressants and outpatient psychotherapy from mental health institutions) for depression is as low as 0.5% (8).

The effectiveness and acceptability of digital CR interventions have been preliminarily verified in recent years (9). However, there is a lack of attention to the challenge faced by individuals experiencing depression in identifying NAT. Existing digital CR interventions elicit emotional arousal mainly through imagination and recollection, a process influenced by factors such as users’ autobiographical memory and imaginative ability. Consequently, evaluating and ensuring the activation of cognitive schema becomes challenging (10), which plays a crucial role in acquisition of CR skills and subsequent efficacy of interventions (11, 12). Current digital CR interventions primarily focus on the evaluation and modification of NAT. The intervention promptly proceeds to the subsequent stage once the user reports the initial NAT they identify, which may unintentionally lead to the omission of other important NAT (13), potentially compromising effectiveness (14).

Virtual Reality (VR) broadly refers to computer-generated interactive environments, providing safe, controllable, and repeatable scenarios (15). Numerous studies have integrated VR with various psychotherapies, and their effectiveness and safety have been preliminarily validated (16–18). VR holds potential advantages in assisting with the identification of NAT in CR for depression. First, VR can trigger more emotional arousal than imaginative approach (10, 19). VR scenarios enable users to acquire perceptual and emotional experiences similar to those in the real world, thereby minimizing the influence of memory, imagination, and experiential avoidance. Also, VR has the potential to create a non-judgmental environment, facilitating the reduction of defense mechanisms in individuals with secondary (or state) alexithymia (3), therefore enhancing their ability to identify and articulate their thoughts and emotions. Moreover, VR is the most concrete form of multimedia, requiring minimal cognitive effort from users, making it suitable for individuals experiencing depression with impaired learning capacity (20). VR also enables activation of visual, auditory, and kinesthetic encoding modes, enhancing the retention of psychological techniques (21). In addition, VR interaction enhances the enjoyment of learning, thereby promoting user engagement and proactivity (22, 23).

However, VR-assisted CR for depression is yet to be explored (24, 25). In 2008, Botella et al. initially employed the EMMA’s World VR system as a sandbox-like tool, to help an individual with complicated grief to express thoughts and emotions (26). In 2021, the study conducted by Kocur et al. developed CAT-DB, an auxiliary tool for CR, in which participants are faced with a virtual avatar expressing their personal dysfunctional beliefs to implement role playing technique (19). That same year, Bolinski et al. developed Zondag Virtual Restaurant as a VR scenario to elicit negative emotions (10). However, in this study, VR was only used as a tool for emotional induction, still requiring the full participation of a psychotherapist to implement CR.

The aim of this study is to evaluate the feasibility of a VR-based self-guided training on identification of NAT (VR-STINAT) for depression, including its efficacy, acceptability and safety, using a mixed methods design primarily within the healthy population. The study posits that the VR-STINAT is effective, safe and acceptable in healthy adults.

## 2 Methods

### 2.1 Research design

A convergent mixed-method design was adopted to investigate the feasibility of the VR-STINAT among healthy individuals (27, 28). Quantitative and qualitative data were collected and analyzed concurrently from the same group of participants to obtain an overview of the user experience, as well as to reveal personal experiences, thereby enhancing understanding and reflection of the training process. The integration of quantitative and qualitative findings was conducted during the interpretation stage. Considering the potential emotional fluctuations that NAT identification training might cause and to avoid potential harm to patients with depression (29), this preliminary exploratory feasibility study targeted only healthy individuals who also experience NAT. This study has been approved by the Ethics Committee of the Shanghai Mental Health Center (2019-51C2), and completed clinical research registration (ChiCTR2000040894). All participants have signed informed consent.

### 2.2 Participants and recruitment

Inclusion criteria for the study included being aged between 18 and 60 years old, being able to read and understand Chinese, scoring below 10 at the Patient Health Questionnaire-9 (PHQ-9) (30). We excluded individuals who suffered from severe physical or mental disorder as assessed with the Mini International Neuropsychiatric Interview Version 4.0.0 (MINI) (31), such as alcohol and/or substance dependence disorder, bipolar disorder, psychotic disorder, suicidal ideation, and those who had received psychological therapy within the past 3 months. The sample size was determined by data saturation, using a saturation table (32). Enrollment stopped when no new themes emerged in the qualitative data.

Participants were recruited online in January 2022. A maximum variation purposive sampling was used to ensure diversity in the sample across gender, age, education level, and experience in VR use. A total of 688 online screening questionnaires were collected initially, out of which 43 samples were obtained. A total of 24 participants completed the offline enrollment screening, out of whom 4 participants were excluded due to bipolar disorder (n=2), post-traumatic stress disorder (n=1), suicidal ideation (n=1), resulting in a final inclusion of 20 participants.

### 2.3 Study procedure

The study was conducted in Shanghai Mental Health Center. After completing baseline assessments, the enrolled participants registered for training accounts and imported their personalized information. Subsequently, they underwent VR-STINAT, followed by post-intervention assessment and semi-structured interview. The entire training process was closely monitored by researchers, who promptly documented any issues that arise during the training. At the training site, recliner chairs and eye patches are provided to alleviate symptoms such as dizziness, nausea, dry eye. The participants were instructed to submit daily homework on NAT identification for 7 days after the training and subsequently complete follow-up questionnaires. The offline study required approximately 2.5 hours, and the online homework took about 15 minutes per day.

### 2.4 VR-based training

The VR-STINAT was iteratively developed with the involvement of end-users (CBT therapists, psychiatrists, patients with depression, and healthy individuals), based on cognitive load theory -learning from worked example (33), and Mayer’s cognitive theory of multimedia learning (34). Gamification elements such as feedback and achievements were incorporated to enhance enjoyment, and engagement (35).

**Figure 1:**
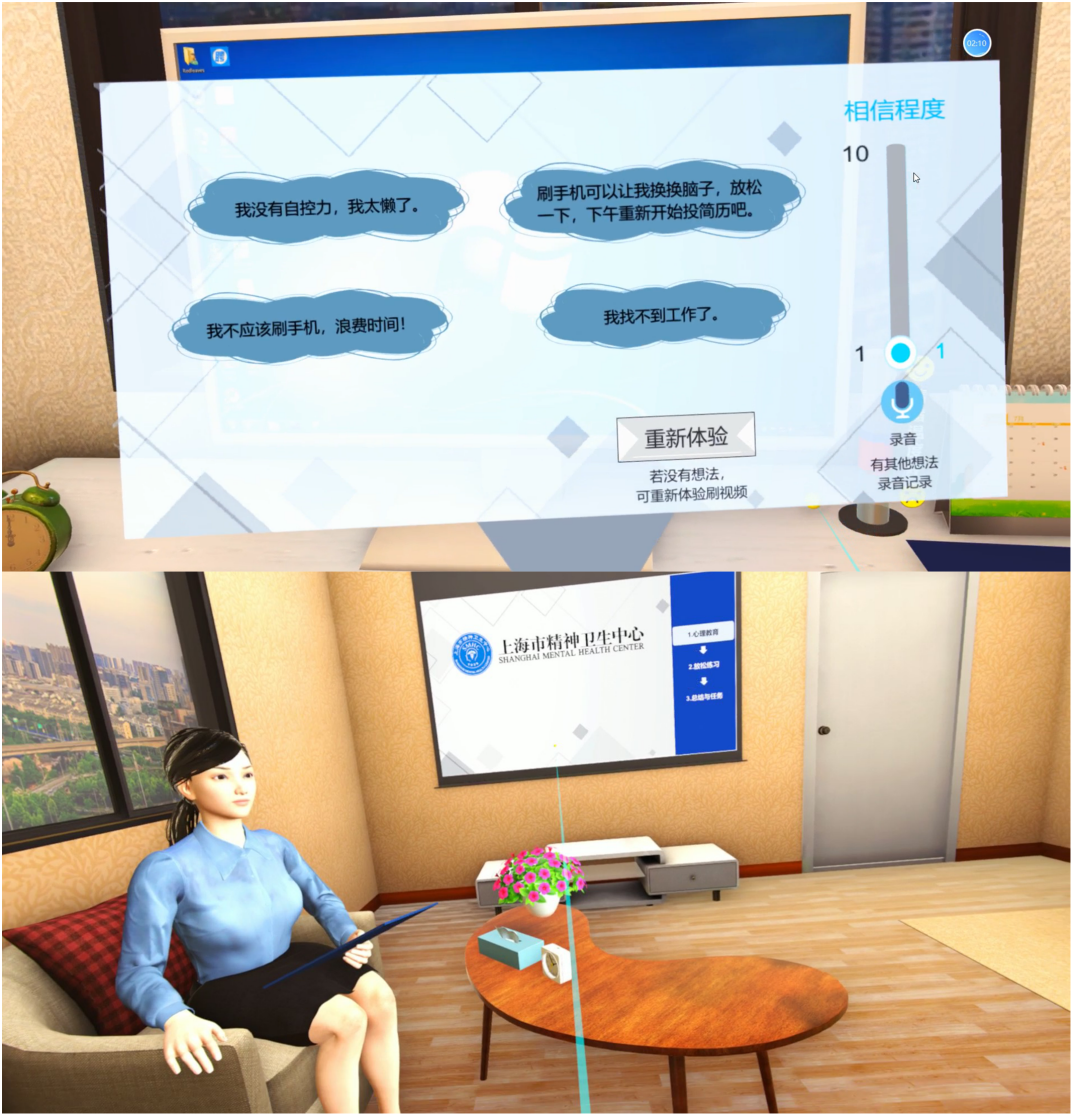
Screenshots of VR-STINAT.

The VR-STINAT includes three modules: ① psychological education, ② NAT identification training in VR scenarios, and ③ practice in personally experienced scenarios (see **Figure 1**). The initial module helped users understand the training process and learn about Beck’s cognitive model through worked-out examples (2). The second module consists of six VR scenarios, including various interactive elements such as mobile phones, during which users were required to complete several tasks of NAT identification. In the third module, the virtual psychotherapist guided users to describe their personal experiences and to identify their NAT and emotions that arose in those particular situations. A fading procedure was implemented in which problem-solving elements are progressively incorporated into example study until the users could solve problems on their own. Identification of AT and emotions was facilitated through an operable panel suspended in virtual space, enabling interaction via controllers and voice input. The first and third modules were implemented within a virtual psychotherapy room, which was modeled based on actual psychotherapy rooms. Participants were given the option to choose the gender of their virtual therapist, whose voices were provided by experienced psychotherapists, and physical appearance resembling that of real psychotherapists. Personalized information of participants (e.g. work experience) was preloaded in the VR scenarios to enhance involvement and emotional arousal.

VR scenarios simulate first-person experiences, capable of evoking a notable level of emotional arousal and providing opportunities for practicing the identification of NAT. In contrast to specific phobias and other mental disorders, the negative cognitive induction scenarios associated with depression lack specificity. The VR scenarios in the VR-STINAT were generated through qualitative research on psychotherapy documentation and homework from our previous CBT intervention studies for depression.

Combining VR with NAT identification may give rise to potential risks, including mood fluctuations, self-harm, or even suicide. To address this, the VR-STINAT has an early warning mechanism that promptly alerts the user’s doctor or psychotherapist when the level of negative emotional arousal exceeds a predetermined threshold. The doctor or psychotherapist then evaluates the user’s risk of suicide and provides preventive measures, including psychological education, contacting family members, and making referrals. Besides, relaxation techniques such as abdominal breathing and progressive muscle relaxation were provided in virtual natural environments following the training to mitigate emotional distress.

Participants entered the VR environment by means of a commercially available head-mounted display (HTC VIVE COSMOS 2Q2R100), connected to a laptop (ALIENWARE x17 R1 17.3-inch 8-core i7 32G 1T RTX3070 165Hz) on which the application was run. The Steam VR platform was used as the intermediary between hardware input and the intervention software (36). Participants interacted with the VR environment using two wireless hand-controllers.

### 2.5 Outcome measures

#### 2.5.1 Quantitative outcomes

##### 2.5.1.1 Effectiveness

The Thought Record Skills Assessment (TRSA) (37, 38) was used to evaluate participants’ mastery of AT identification by rating their homework based on the content of situational events, emotions, and AT. Two researchers (YBX and SBB) independently assessed the scores and engaged in discussions to achieve consensus in case of any discrepancies.

An adapted version of Cognitive Therapy Awareness Scale (CTAS) was used to assess the participants’ knowledge of CR theory and application. The original CTAS consists of 40 true or false questions (Cronbach α=0.58). We selected 12 items related to automatic thoughts (37).

##### 2.5.1.2 Safety

The Chinese version of Simulator Sickness Questionnaire (SSQ) was used to evaluate physiological safety (39). In terms of psychological safety, emotional arousal was assessed via self-report through the operable panel in VR scenarios, and emotional intensity was assessed via the Visual Analogue Scale (40) (1=almost none, 10=extremely). VR scenarios where the intensity of negative emotions reached 8 were marked.

##### 2.5.1.3 Acceptability

The acceptability of the VR-STINAT was assessed according to the Virtual Reality Hardware Acceptance Model (VR-HAM) (41), including four dimensions: perceived usefulness, perceived ease of use, attitude towards use, and intention to use. The duration of VR training was recorded.

##### 2.5.1.4 Engagement and adherence

Engagement was measured by the number of exercises participants voluntarily performed in the third module of VR-STINAT. Adherence was measured by the participants’ completion of assigned homework.

##### 2.5.1.5 Depressive symptoms

Depressive symptoms were assessed using the Chinese version of Patient Health Questionnaire Depression Scale (PHQ-9), a self-report questionnaire for the assessment of depressive symptoms (30).

##### 2.5.1.6 Presence

Immersion was assessed using the Chinese version of the Igroup Presence Questionnaire (IPQ), a 13-item self-reported questionnaire rated on a 7-point scale. The items are categorized into three dimensions: spatial presence, involvement, and realness (42).

#### 2.5.2 Qualitative outcomes

A semi-structured interview questionnaire was developed to understand the participants’ experience with the VR-STINAT (43, 44). The interview included a series of open-ended questions, covering the following topics: expectations, experience, opinions and suggestions (**Supplementary Material 1**). Before the interview, we obtained participants’ informed consent to record audio. The interview was conducted in a quiet and private environment, ranged between 8 and 35 minutes (Mean=14 minutes). Each interview was fully recorded on a voice recorder and transcribed verbatim. Furthermore, feedback on the overall experience of VR and homework was collected 7 days after the VR intervention to ensure thorough gathering of participants’ feedback.

### 2.6 Data Analysis

Quantitative data analysis was conducted using SPSS V.28.0. Descriptive statistics for categorical variables and safety data were reported as frequencies and percentages. Depending on the data distribution, continuous variables were reported as mean ± standard deviation (M ± SD) (for normal distribution), and as median (interquartile range) (for abnormal distribution). Pearson correlation with Bonferroni correction was used for correlation analysis. T-tests or non-parametric tests were used for continuous data, while chi-square tests were used for categorical data. When the minimum expected value is below 5, Fisher’s exact test was applied. When analyzing the pre-post differences of PHQ-9, a repeated measures analysis of variance was used. The significance level was set at α = 0.05.

Qualitative material was analyzed through the six-step approach (45) to thematic analysis using nVivo (V.12). The thematic analysis was conducted with guidance from the Technology Acceptance Model (TAM) and the Theory of Planned Behavior (TPB) (46, 47). All transcripts were read and coded individually by two researchers (YBX and SBB). They then discussed their codes and generated themes in an iterative process, referring back to the transcripts and the original codes to ensure that the analysis was representative of the data.

## 3 Results

For sociodemographic characteristics of participants, see **Table 1**.

**Table 1:** Sociodemographic and baseline characteristics.

ID	Age (Years)	Gender	Highest educational level	Previous experience in VR use	PHQ-9
1	26	M	University	1-4 times	4
2	28	M	University	1-4 times	9
3	41	F	Vocational education	0 times	6
4	35	M	Postgraduate	1-4 times	0
5	32	F	University	0 times	1
6	32	F	University	1-4 times	1
7	39	F	Postgraduate	5-9 times	3
8	34	M	University	1-4 times	0
9	38	F	Vocational education	0 times	4
10	44	M	University	1-4 times	3
11	31	F	Vocational education	0 times	4
12	31	M	Postgraduate	5-9 times	7
13	29	M	University	0 times	3
14	27	M	Postgraduate	1-4 times	0
15	35	F	Postgraduate	0 times	3
16	31	F	Postgraduate	1-4 times	1
17	33	M	University	0 times	0
18	30	F	University	0 times	7
19	23	F	University	0 times	4
20	27	F	University	0 times	9

### 3.1 Quantitative results

All participants (n=20) completed the training and post-training assessment. **Table 2** summarizes the quantitative results.

**Table 2:** Outcome measures.

	PHQ-9<5 (n=15)	PHQ-9≥5 (n=5)	Total (n=20)	*t/U/χ²*	*p*
Effectiveness					
TRSA average score	1.84 ± 0.15	1.88 ± 0.12	1.85 ± 0.14	-0.43	0.673
TRSA NAT score	1.79 ± 0.34	1.77 ± 0.29	1.78 ± 0.32	0.09	0.927
CTAS correctness	76.11% ± 15.71%	78.33% ± 16.25%	76.67% ± 15.44%	-0.27	0.789
Engagement and adherence					
Homework completion rate	98.10% ± 5.03%	91.43% ± 12.78%	96.43% ± 7.86%	1.14	0.313
Number of exercises	7.07 ± 0.70	7.60 ± 2.70	7.20 ± 1.40	-0.44	0.684
Number of exercises in training	1.47 ± 0.64	1.40 ± 0.89	1.45 ± 0.69	0.18	0.857
Acceptability	83.03% ± 10.52%	73.64% ± 3.05%	80.68% ± 10.04%	3.09	0.006
Perceived usefulness	3.40 ± 0.51	3.13 ± 0.30	3.33 ± 0.47	1.43	0.179
Perceived ease of use	3.22 ± 0.57	3.00 ± 0.24	3.17 ± 0.51	1.22	0.238
Attitudes toward use	3.44 ± 0.41	3.00 ± 0.41	3.33 ± 0.44	2.10	0.051
Intention to use	3.17 ± 0.75	2.50 ± 0.50	3.00 ± 0.74	1.84	0.082
Completion Time	67.07 ± 12.28	63.00 ± 11.89	66.21 ± 11.99	0.59	0.562
SSQ	0.00(7.48)	0.00(69.19)	0.00(10.29)	28.50	0.333
IPQ	54.93 ± 10.11	47.20 ± 7.12	53.00 ± 9.89	1.57	0.133
Emotional tolerance	13.33%	20.00%	15.00%		1.000
△PHQ-9	-0.93 ± 2.12	-3.00 ± 7.28	-1.45 ± 3.91	0.63	0.564

PHQ-9, 9-item Patient Health Questionnaire Depression Scale; TRSA, Thought Record Skills Assessment; CTAS, Cognitive Therapy Awareness Scale; VR, Virtual Reality.

The TRSA scores for homework showed a high inter-rater reliability of 82.64% (357/432). According to SSQ, the majority of participants (n=18, 90%) experienced negligible symptoms (SSQ score <5), while two participants (10%) reported problematic symptoms (SSQ score >20) of moderate general discomfort, eyestrain, difficulty focusing, fullness of head, nausea, dizzy (eyes open), and moderate-to-severe fatigue, blurred vision. The most common adverse event was fatigue (n=5), and the most frequently observed adverse event of moderate to severe intensity was craniofacial pain induced by VR glasses (n=3, 15%). Three participants (15%) experienced relatively intense negative emotions.

Subgroup analysis was conducted between the participants with baseline PHQ-9 scores above and below 5. The VR-STINAT was significantly more acceptable for the PHQ-9<5 group (t = 3.09, p = 0.006). The mean scores of attitudes toward use and intention to use between two groups were to the edge of significantly different.

Correlation analysis showed that pre- and post-training change in PHQ-9 score (△PHQ9) was positively correlated with age (r = 0.493, p = 0.032), indicating that improvement in depressive symptoms decreases with increasing age. Repeated-measures ANOVA with PHQ-9 score as dependent variable and age as covariate revealed a significant decrease in depressive symptoms after controlling for the effects of age (F = 7.135, p = 0.016).

### 3.2 Qualitative results

**Figure 2:**
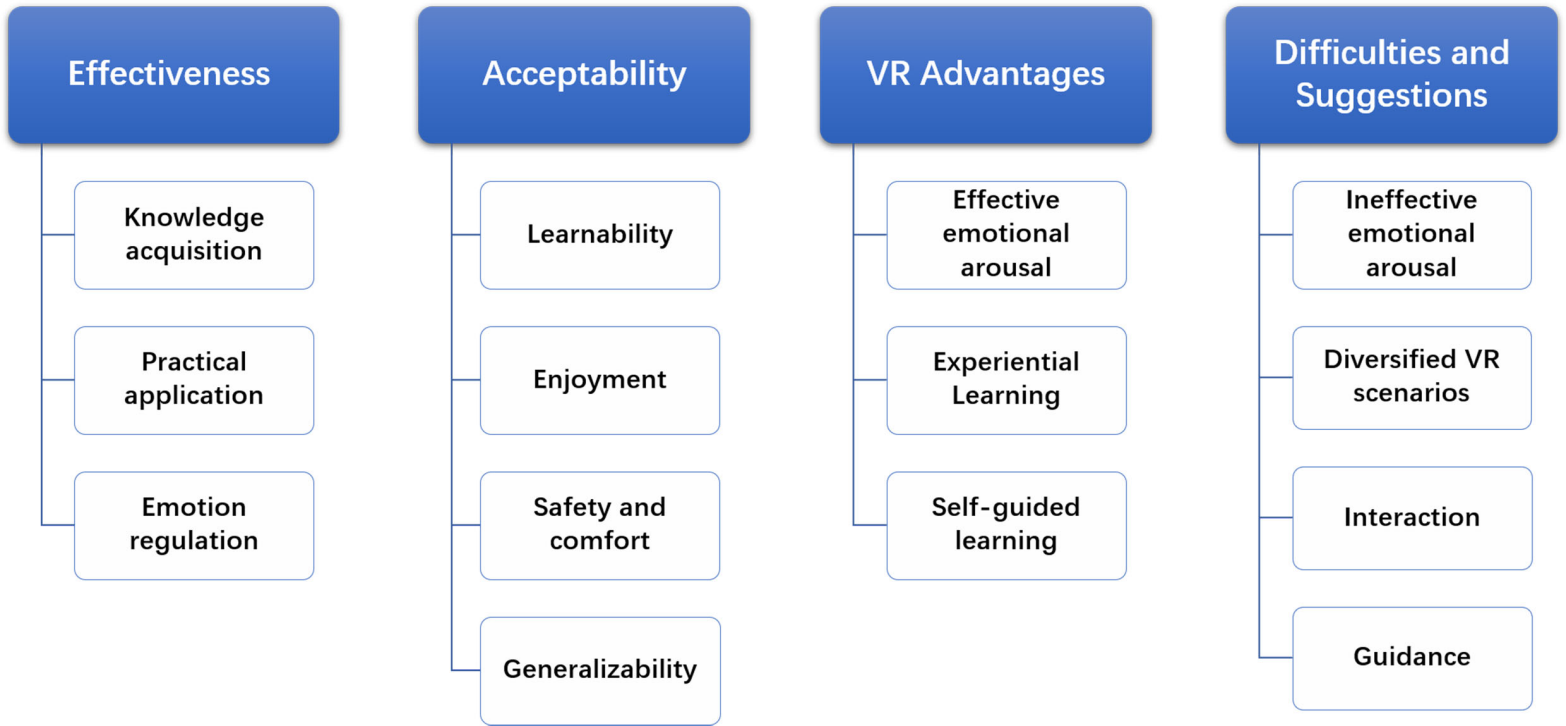
Structure of the thematic analysis.

Four core themes emerged through thematic analysis: effectiveness, acceptability, advantages of VR, difficulties and suggestions for improvement (see **Figure 2**). Any themes mentioned by at least two participants (10% of the sample) are depicted.

#### 3.2.1 Effectiveness

The majority of participants (n=17) reported that through VR-STINAT they have learned how to identify their NAT and emotions, with awareness of the impact of thoughts on emotions, and have realized the need to distinguish between subjective perceptions and objective facts. Some participants (n=13) made further associations that emotions can be regulated by adjusting cognitions and started to summarize and reflect on the identified NAT.

*“I’ve learned a method. When you want to analyze something, the thought record can help you calmly analyze your thoughts at that moment. You can see some mood swings and score your mood.” (P01)*

Participants also found that VR-STINAT positively influenced their application of the skills (n=16), by enhancing their self-efficacy and an awareness of continuous practice. Several participants (n=8) proactively planned to continuously practice the learned psychological skill in their daily lives.

*“I truly learned the skill to regulate emotions through cognition … When encountering situations in the future, I can slow down a bit, think about which of my thoughts caused the emotion, and further evaluate whether my thoughts are objective and accurate. This can reduce some unnecessary emotional consumption.” (P05)*

After one week of practice, participants (n=9) reported that identifying NAT helped regulate their emotions and fostered greater acceptance and compassion towards their emotions, providing an enhanced understanding of their internal experiences. Participants reported that when they experienced negative emotions, they learned not to dwell on the negativity but instead to start using the psychological techniques to analyze (n=3).

*“Trying to adjust my automatic thoughts is helpful for problem-solving and mood adjustment.” (P11)*

*“This VR experience made me realize that all thoughts are actually normal. Everyone has their automatic thoughts, which then generate various emotions, good and bad.” (P13)*

*“I will not indulge in negative emotions; instead, I can step back and analyze the situation.” (P15)*

#### 3.2.2 Acceptability

Most participants found VR-STINAT easy to understand and use (n=12), as well as engaging (n=18). Participants perceived the main source of enjoyment in VR-STINAT to be its immersive and gamified experience, role-playing and human-computer interaction. They also believed that it had the potential for broader application (n=6), serving not only healthy individuals but also patients with depression who feel ashamed due to illness or have difficulty accessing treatment.

*“I had no prior experience in VR, so overall this experience was very good. The operation was quite convenient, and even though I’m not very good with electronic devices, I found it completely manageable.” (P05)*

*“It was quite interesting and somewhat immersive … I even reached out with the controller to see if there was a real table in front of me, and I wanted to give a thumbs-up on my social media in VR. It was quite fun.” (P10)*

*“It felt as if I were playing a game or something. It’s very interesting.” (P19)*

*“I know that psychological therapy is not that accessible right now, and people with mental conditions are often reluctant to seek help. If this could be used at home, it would be helpful.” (P09)*

Some participants reported feeling fatigued and uncomfortable after using the head-mounted virtual glasses for extended periods (n=4).

*The thing with VR glasses is that they’re not very user-friendly. They can get pretty heavy and start to feel uncomfortable if you wear them for a long time. ( P02)*

#### 3.2.3 VR advantages

Most participants stated that VR-STINAT allowed them to immerse themselves in a first-person perspective, effectively evoking their emotional experiences, which helped them practice identifying their AT (n=10). They reported that certain components within the system could help them alleviate the negative emotions aroused (n=3).

*“It really evoked my emotions. At that moment, I felt depressed … This sense of frustration was very clear to me. So, the overall environment, including the scenarios’ immersion, was quite strong. I didn’t like the negative emotions, but I think this method worked very well. Although it induced negative emotions, it made the learning experience more impactful because my emotions were triggered.” (P07)*

Participants mentioned that, after theoretical learning, immediate practice of the learned psychological skill in VR scenarios was a concrete and step-by-step way to help them apply these techniques in real life (n=7). The participants reported that compared to computer-based systems, this VR-based system is more engaging, and the use of head-mounted virtual goggles effectively isolates distractions from the environment, enhancing their focus during training (n=2).

*“It allowed me to better understand how to analyze or use this skill in an immersive way. Simply having the knowledge and immediately analyzing it on my own might not be that effective.” (P10)*

*With computer, there are many external interferences that make it difficult to concentrate. However, these VR glasses provided a fully immersive experience, you can truly immerse yourself and focus on your own feelings in that moment. (P06)*

Some participants mentioned that the self-guided format of VR-STINAT, which does not require the actual presence of a psychotherapist, allowed them to feel more relaxed and at ease, and to experience self-directed learning (n=2).

*“VR is more comfortable and freer because it doesn’t involve facing a real person. It allows for more freedom and relaxation.” (P19)*

#### 3.2.4 Difficulties and suggestions for improvement

Participants encountered several difficulties while using VR-STINAT, including experiencing multiple virtual training scenarios in a short time, which made it difficult for their emotional responses to keep up (n=2), and finding that the VR scenarios did not match their current mood, thus failing to evoke emotional responses effectively (n=2).

*“My emotions couldn’t keep up because I experienced a few days of life in just a few minutes.” (P01)*

*“It felt like watching a story … I found that it didn’t match my current mood very well.” (P20)*

Participants also provided suggestions for improving the system, including enriching the existing VR scenarios and providing more options (n=7), increasing the quantity, sensitivity, accuracy, and variety of human-computer interactions (n=5), and offering more personalized design in the training process and guidance (n=5).

*“The scenes and storylines could be more diverse.” (P02)*

*“There could be more interaction, such as interaction with the virtual therapist.” (P18)*

*“The interaction was a bit slow sometimes, and the operation needs to be more responsive.” (P03)*

*“It would be way easier if I could control it just by speaking.” (P06)*

*“It would be better if the first part were more detailed, with more examples and analyses. People with stronger comprehension skills might be fine, but those with weaker comprehension might find it difficult to understand.” (P17)*

## 4 Discussion

To the best of our knowledge, this preliminary mixed-methods study represents the first attempt to investigate the feasibility of a VR-based self-guided training on the identification of NAT for depression. The effectiveness, safety, and acceptability of VR-STINAT were found to be favorable among the healthy population. This study addresses the limitations of NAT identification training in existing digital CR interventions, expanding the scope of VR-assisted psychotherapy for depression.

### 4.1 Effectiveness

Our quantitative and qualitative data both indicate the effectiveness of VR-STINAT in training participants to identify their NAT. After training, participants demonstrated a higher level of proficiency in identifying NAT in their homework and a better understanding of cognitive theory, surpassing previous research on APP intervention conducted in non-clinical populations (13). Participants reported that VR-STINAT facilitated their acquisition and utilization of NAT identification technique, as well as enhanced their awareness of the impact of AT on emotions following an event. To date, no other studies have employed VR for self-guided training aimed at identification of NAT associated with depression. Our research fills this gap in the literature.

Aligned with the theory of situated learning (48), participants perceived this system as facilitating contextualized learning and experiential practice, making abstract theoretical knowledge more concrete, thereby enhancing the practical application of the technology. Secondly, participants mentioned that the VR scenarios in this training effectively elicit their emotional responses. Emotional arousal indicates the activation of cognitive schemas, which is a prerequisite for identification of AT and plays a pivotal role in facilitating the acquisition of CR techniques and reaching the desired outcomes (11, 12). In addition, the completely self-guided training format offers participants a non-judgmental and secure environment conducive to the expression of their authentic NAT rather than processed ones (6).

The homework compliance of this study was higher compared to previous studies that implemented CBT interventions digitally, face-to-face, or via telephone (49–52). Several participants proactively planned to continue practicing the NAT identification skill in real-life scenarios even before the homework was assigned. The challenge of identifying scenarios that necessitate the use of CR techniques is a prevalent factor contributing to clients’ discontinuation of utilization (53). VR-STINAT provides a range of typical scenarios that can induce NAT, thereby facilitating the participants’ acquisition of knowledge on when and how to employ this technique. Following the training, several participants summarized the potential application scenarios for this technique. Furthermore, the training necessitated active participation from the participants, thereby fostering an enhanced self-efficacy and sense of accomplishment, consequently resulting in increased skill use quantity and frequency (54).

### 4.2 Acceptability and safety

The VR-STINAT demonstrated high acceptance rates among the healthy population. The findings of this study align with previous studies combining VR with psychotherapies (55, 56). Most participants reported the interface of VR-STINAT to be clear, concise, user-friendly, and easy to learn, demonstrating a high level of usability. Most participants showed a positive attitude towards the use of VR-STINAT, considering it novel and interesting, as well as beneficial for mental health. They also perceived VR-STINAT as a professionally designed and scientifically grounded approach, suitable for both healthy individuals and those at high risk, thereby indicating a certain level of intention to recommend.

Subgroup analysis revealed that participants with depressive symptoms exhibited significantly lower acceptability of this system compared to those without depressive symptoms. The discrepancy may reside in attitude toward using and intention to use, while no subgroup disparity was observed in perceived usefulness and perceived ease of use. Qualitative interviews revealed that participants with depressive symptoms may potentially experience heightened negative emotions within the VR scenarios, or they may encounter difficulties in fully immersing themselves in the VR scenarios due to an excess of negative thoughts of themselves, leading to a relatively unfavorable disposition towards the VR-STINAT.

The physiological safety of the VR-STINAT was comparable to that observed in previous studies on VR (57). The most common adverse reactions were fatigue and tenderness in the head and face, which can be attributed to the VR headset and prolonged training duration. No participant discontinued the use of VR-STINAT due to adverse reactions, and reported discomforts were effectively alleviated following a 15-20 minute session using a recliner chair and eye patches for relieving eye fatigue. Among the two participants who reported problematic symptoms, one had recently completed a full day of work, suggesting that their discomfort might not be unrelated to their pre-intervention state. Therefore, future training sessions should be scheduled to avoid times when participants may be experiencing discomfort. Additionally, it is recommended to record SSQ scores as a baseline measure prior to the intervention.

A few participants reported intense experience of emotional distress. Previous studies have suggested that activation of negative cognition may result in a transient exacerbation of depressive symptoms, and this temporary deterioration is positively associated with treatment outcomes upon completion (29). Additional psychological techniques are still necessary to address the negative emotions experienced by participants post-training. Therefore, VR-STINAT may serve as a supplementary tool for CR intervention.

### 4.3 Suggestions for improvement

The VR-STINAT still has potential for further improvement. First, participants proposed an increase in the availability of diverse VR scenarios for their selection. The current VR scenarios primarily emphasize achievement-related events, rendering them particularly suitable for autonomous individuals. Conversely, sociotropic individuals may exhibit a greater propensity to experience emotional reactions and NAT triggered by negative interpersonal events (58).

Furthermore, the participants proposed that the richness and comfort of human-computer interaction can be further enhanced, for instance, through voice command. In addition, regarding training process and guidance, some participants exhibited a preference for more comprehensive instructions, while others leaned towards a more succinct approach. Therefore, it may be worth considering the division and refinement of modules within the training to offer a personalized training experience.

Moreover, participants mentioned the challenge of synchronizing emotional reactions when exposed to multiple VR scenarios within a limited timeframe, stemming from the temporal inconsistency between the VR environment and the real world. The factor of time should be taken into account when constructing VR scenarios in the future.

### 4.4 Symptoms of depression

The results of our quantitative analysis suggest that, after controlling for age, the VR-STINAT effectively mitigates depressive symptoms. Participants reported that the training and post-training homework facilitated their comprehension of emotions and NAT, promoted cognitive reflection, and fostered greater self-acceptance of both themselves and their emotions.

Previous studies have shown that self-monitoring of emotions and thoughts can foster adaptive self-attention and self-awareness, augment self-efficacy, subsequently contributing to the amelioration of depression (59). Previous studies that employed VR to alleviate depression were predominantly based on Erikson’s psychological theory, mindfulness, exercise, and behavioral activation [24]. However, no research has yet explored the potential of integrating NAT identification technique within VR for depression (25).

### 4.5 Limitations and future research

Several limitations should be considered when interpreting the results. First, this study is a small exploratory feasibility study. In consideration of ethical concerns pertaining to the safety of emerging technologies, this study specifically targeted a sample of healthy individuals who also experience NAT, thereby constraining the generalizability of the findings to clinical population. The feasibility and effectiveness of VR-STINAT in patients with depression have been assessed through a subsequent randomized controlled trial conducted in a larger sample, and the findings will be independently published in another article (Yang YQ, et al. Self-Service Virtual Reality-Based Psychotherapy for the Treatment of Mild to Moderate Depressive Disorders: A Randomized Controlled Trial). Secondly, the construction of VR scenarios in this system was based on the NAT-evoking scenarios reported by patients with depression in our previous studies. However, the representativeness of these scenarios in the depressed population remains unverified and necessitates further studies to establish their reliability and validity. Finally, future research should integrate VR-STINAT as an auxiliary tool into CR interventions to evaluate its effectiveness and cost-efficiency among individuals with depression.

This study represents the first attempt to employ VR for delivering self-guided training on identification of NAT, and to explore its feasibility through a mixed-methods approach. The findings indicate that the VR-STINAT demonstrated high effectiveness, acceptability, and safety among healthy individuals, showing potential as an auxiliary tool for CR interventions. This study enriches the research on VR-assisted CR interventions for depression and offers valuable insights for exploring novel models of CR interventions for depression. CR is one of the key psychological techniques for depression, and future research on VR-based CR interventions holds potential for enhancing the efficacy and accessibility of mental health services for depression.

## Data Availability

The raw data supporting the conclusions of this article will be made available by the authors, without undue reservation.
