# Supplementary material for: A virtual reality-based self-guided training on identification of negative automatic thoughts in healthy adults: a mixed-methods feasibility study
Source: Front Psychiatry. 2024 Nov 27;15:1479207. doi: 10.3389/fpsyt.2024.1479207 (PMC11632459; doi:10.3389/fpsyt.2024.1479207)
Supplement: Supplementary file 1 [file DataSheet1.pdf]

Supplementary Material 1

Interview Schedule

1. What were your expectations for this system before you use it?

2. How was your experience of using VR-STINAT?

（1）Was it easy to use?

（2）Was the software engaging/inviting/interesting?

（3）Did the system meet your expectations?

（4）Were there any features that you liked at lot?

（5）Were there any features that you did not like?

3. Have you found your experience with VR-STINAT (un)helpful?

（1）Have you learned anything after using VR-STINAT? What?

（2）Were there any specific features that were useful? What were they?

（3）Was there any instance where VR-STINAT was unhelpful?

4. What are your thoughts on the modules of VR-STINAT?

5. Do you have any questions or is there anything else you would like to add?
